# Application of layers of protection analysis to prevent coronavirus infection

**DOI:** 10.1002/prs.12362

**Published:** 2022-03-31

**Authors:** Ali Mokhber, Shivani Aggarwal, Pablo García‐Triñanes

**Affiliations:** ^1^ Materials and Chemical Engineering Group, School of Engineering University of Greenwich Medway UK

**Keywords:** COVID fatality index, COVID‐19 protection layer health protocols, layers of protection analysis, probability of failure on demand, transmission rate

## Abstract

Layers of protection analysis (LOPA) methodology is applied to an encounter with the SARS‐COV‐2 infection as an initiating event, and subsequently, independent protection layers (IPLs) (namely health safeguarding protocols), such as social distancing, ventilation, hand hygiene, face masks, and vaccinations. LOPA is applied considering numerical quantification of the COVID fatality index in order to manage the transmission risk to a tolerable level, namely the fatality risk due to seasonal flu. This measurement tool quantifies the ratio of the annual death rate due to the SARS‐COV‐2 infection to the annual death rate of the common flu, and it is applied to a chemical plant. The lower this quantified value is, the more the COVID‐19 infection death rate approaches that of the common flu. Thus, any improvement in safeguarding protocols should reduce this index. The input data is based on public domain COVID‐19 infection statistical data and websites accessible in the United Kingdom. The COVID‐19 transmission rate is statistically analyzed with random number sampling to simulate the random pattern of the virus' person‐to‐person infection in the community. The success of the COVID‐19 protection protocols is probabilistic and depends on the public's compliance, which is modeled by observational surveys.

## 1 INTRODUCTION

From a chemical engineering point of view, the transmission of the SARS‐COV‐2 virus is a process, and the disease COVID‐19 can be managed like any other process hazard. Considering that layers of protection analysis (LOPA) methodology has successfully been applied to safeguarding process plants, it can also perform risk assessments to evaluate the relative probabilities of virus transmission, infection, and death. This is vital moving forward due to the high transmission rates of the SARS‐COV‐2 virus and its severe consequences. Lockdowns and heavy restrictions were imposed to help limit the SARS‐COV‐2 contagion spreading; however, in order to make life sustainable with these restrictions, new methods/standards need to be incorporated into day‐to‐day life to help manage this virus and guarantee business continuity.^1^, ^2^, ^3^

LOPA is a semi‐quantitative risk assessment methodology and well documented in literature sources.^4^, ^5^, ^6^ LOPA requires three main inputs: “Risk Tolerability Criteria,” “Initiating Event Frequency,” and “Probability of Failure on Demand (PFD)” for IPLs. For the COVID‐19 case, the risk tolerability criteria are taken as a comparison of the annual frequency of death due to COVID‐19 and for seasonal flu.

### 1.1 Literature review on health protocols

The public domain information on the compliance of the health protocols identifies the driving features of the rules.

A guidance written by Public Health England^7^ notes COVID‐19 care pathways and sets governance and responsibilities for stakeholders and the public to manage the infection risk. It sets objectives for infection control and transmission precautions, use of personal protection equipment, patient care, and the risk of respiratory infection transmissions.

Greenhalgh et al.^8^ present evidence and guidelines for the use of face masks. The key message from the paper states that we should sometimes act without definitive evidence as a precaution. There have been widespread debates as to the effectiveness of the use of face masks; however, it can be agreed that even limited protection is beneficial and could prevent some transmission of this disease, which, in turn, would save lives due to COVID‐19 being such a serious threat. Therefore, wearing masks in public should be advised. The World Health Organization also provides guidelines on the use of face masks for children and adults.^9^

A report written by the European Centre for Disease Prevention and Control defines targets and instructions for the use of face masks and hand hygiene methods.^10^ Increases in the encouragement of hand hygiene is also recommended in the following reports and have been supported with scientific facts.^11^, ^12^ The latter reference states that when hand washing is carried out, it is essential to limit skin damage by the use of a moisturizer each time the hands are washed.

Research and investigations have been conducted on whether there is an association between hand hygiene and COVID‐19 transmission.^13^ Their conclusion is as follows; in a population‐based sample of Polish adolescents, individuals from regions of low COVID‐19 morbidity presented more beneficial hand hygiene habits than those from regions of high COVID‐19 morbidity.

The origin of the 2 m safe distancing rule has been investigated by Jones et al.,^14^ and the authors concluded that investigations referring to “safe distancing” started in late nineteenth century. Despite limitations in the accuracy of these early study designs, especially for longer ranges, the observation of large droplets falling close to a host reinforced and further entrenched the assumed scientific basis of the 1–2 m distancing rule. Computational fluid dynamics simulations have been used by Blocken et al.^15^ in order to model the safe distancing for people standing still (1.5 m), walking (5 m), and running (10 m).

## 2 COVID RISK MANAGEMENT WITH LOPA METHODOLOGY

The COVID‐19 pandemic can be viewed in the same way as a typical process hazard. It is possible to apply LOPA to the issue of the virus transmission in particular settings, its likelihood of transmission considering layers of protection, or indeed multiplication, and its impact upon individuals, taking into account their demographics and state of health.

The initiating event is the frequency of encountering a person infected with the virus, and the IPLs are social distancing, free air movement (ventilation and open space), face masks, hand hygiene and the vaccine efficacy. These safeguards are collectively referred to as COVID‐19 protection layer health protocols. For LOPA modeling, IPLs are used as barriers to the spread of the virus with their probability of failure on demand (PFD). A “PFD” is a probability between 0 and 1.0, with 1.0 indicating no IPL is present or 100% failure, decreasing as the probability of failure of an element decreases. The calculation does not consider common cause failure of human noncompliance with health protocols.

The input data for LOPA modeling and calculations are based on regional statistical COVID‐19 infection rates and fatalities obtained from websites in the United Kingdom.

### 2.1 Basis of COVID transmission rate

COVID‐19 is atypical of process hazards as it is all‐pervasive, often carried by asymptomatic individuals, without any obvious sign of infection. However, it is possible to evaluate the frequency of an “initiating event” defined as an “effective” contact with an infected person or the transmission rate, based on the following inputs:

1. Local rolling infection rates, for example, those published in the UK as the COVID‐19 virus interactive map for England^16^ and BBC COVID‐19 in the UK^17^
2. Hours spent in the risk area with the potential of person‐to‐person infection
3. Number of human contact events per year with potential virus transmission
4. Adjust for testing and for asymptomatic cases.

These factors are used to evaluate the number of effective infections per year, that is, the transmission rate, which is the initiating event in the LOPA calculation.

#### 2.1.1 COVID testing regime and asymptomatic infection modeling

The COVID‐19 testing regimes are as follows:

- Polymerase chain reaction (PCR) tests are sent away to a lab to diagnose the disease.
- Lateral flow tests (LFTs) can diagnose COVID‐19 within 30 min of taking a sample but are not as accurate as PCR tests.
- Antibody (or serology) tests cannot diagnose active infection but can help to indicate if a person has immunity to COVID‐19.

In this study, LFTs are used to account for the worst‐case scenario for person‐to‐person infection. According to Mahase et al.,^18^ studies have shown that, while false positives are rare with the commonly used lateral flow test, false negatives are much more common. Three results from Public Health England showed that the test's overall sensitivity was 76.8%, meaning that 23.2% were false negatives. Sensitivity dropped to just 57.5% when carried out by self‐trained staff at a track and trace center.

The pooled estimate of the asymptomatic portion of COVID‐19 is 28%, which was used to calculate the transmission frequency.^19^ PCR testing is more accurate than LFT and can be an input if required. Pooled analysis of 16 studies (3818 patients) estimated a sensitivity of 87.8%.^20^

### 2.2 Basis of LOPA modeling

Two parallel infection pathways have been identified: direct transmission from an infected person to the target individual via droplets and aerosols carrying the virus, or indirect transmission, where infected droplets land on a surface,^21^ and are then picked up by the target person and transferred to the soft tissues. These infected droplets can last on surfaces for long periods of time (ranging between 12 h to 2 days).^22^

The calculation is done as a typical LOPA, with the probability of failure on demand of each of the assigned protection layers, such as distancing, face masks, hand sanitization and ventilation; calculating through to a probability of infection for each pathway, summed up to a total probability for all pathways.

The impact of infection on the death rate of infected persons is obtained from the Association of Local Authority Medical Advisors (ALAMA) calculator,^23^ which, given inputs on the age, sex, ethnicity, body mass index (BMI), and various comorbidities, then indicates the probability of death of an infected person. This can be run for typical and vulnerable individuals. This then enables us to compare the probability of death from COVID‐19 to that from seasonal flu and to identify an improvement factor as a target to attain this. The improvement factor is calculated as an index with a numerical value and referred to as COVID fatality index.

For comparison and benchmarking purposes, a COVID fatality Index is introduced in the calculation, which determines the improvement measures in testing regimes and IPL compliance to bring down the cases relating COVID‐19 to seasonal flu fatality level.

## 3 STATISTICAL ANALYSIS

The transmission rate is the initiating event for the LOPA modeling. This rate is based on the person‐to‐person infection rate.

The infection rate is evaluated by statistical regression analysis that is used to look at the correlation between the dependent and independent variables. This method aims to explain the dependent variables in terms of the independent variables through a mathematical relationship, to obtain a prediction of one variable given the value of the other. The regression analysis was done using spreadsheet calculation modules.

The Regression model is being used here to help look at the mean infection rates of COVID‐19 in different locations, to then mathematically simulate the way that the virus infection is spreading among the population. Thus, the input data was randomly selected and fed into a regression analysis model by an analyst; however, in the future, it is envisaged to use a computer software program for the random selection process.

The COVID‐19 infection is unpredictable and could be arbitrary. It is therefore required to describe and predict how the virus transmits itself in the community. Data collection and sampling with statistical models can predict the virus propagation. The infection is also random, which means it is impossible to predict future human infections based on past or present ones. The modeling, therefore, requires probabilistic assessment to account for the randomness. The statistical modeling algorithm uses an “arbitrary random population sampling” approach that is meant to randomize the virus' person‐to‐person transmission in the community. The mathematical calculations are designed to simulate the real‐life virus transmission randomness and develop predictive tools on virus behavior in a given population sample.

For calculation of the infection rate, statistical modeling was performed on a hypothetical Chemical Plant in the United Kingdom.

Once the data was collected and each case rate was randomly assigned to a population sample, a scatter graph was produced, and then for each case, a regression analysis was performed to obtain the straight‐line regression equation.

The geometric mean of the sampling population was used as the numbers in these series are not independent of each other, and in some cases, the numbers tend to make large fluctuations. The calculated geometric mean from the values of the population sample was then inputted into the equation of the regression line that was obtained from the scatter graph. This then calculated the “*y*” value, showing the geometrically adjusted mean infection rate.

The interactive map^16^ and BBC COVID‐19 in the UK website^17^ were useful tools in obtaining information, such as the case rate per 100,000 people, the total number of cases in a given area, and also the rate of change in percentage from the previous week.

## 4 BASIS OF INFECTION RATE CALCULATION IN A CHEMICAL PLANT

**FIGURE 1:**
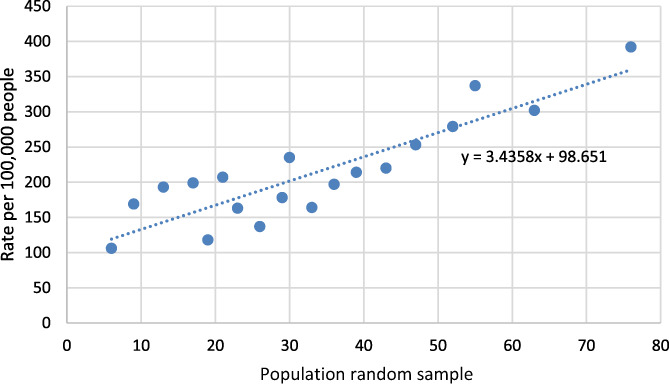
Rate of COVID‐19 cases in typical areas around a Chemical Plant (June 21, 2021)

**FIGURE 2:**
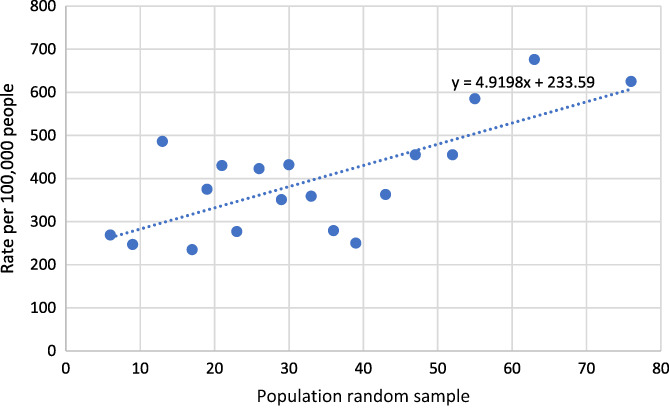
Rate of COVID‐19 cases in typical areas around a Chemical Plant (June 28, 2021)

For the case of the Chemical Plant in the United Kingdom, infection data from the urban municipalities surrounding the site were used. The population of the plant is assumed to be 637, and it is surrounded by 19 local boroughs that are of a reasonable commuting distance; therefore, 19 arbitrary samples were randomly taken. Tables 1 and 2 show the “local home addresses,” “random population sample,” and the corresponding “infection/case rate per 100,000 people.” The sample and infection rate are inputted into the regression analysis for the last 2 weeks of June 2021. It is noted that while the “random population sample” is arbitrary selected, the infection/case rate per 100,000 people is not, instead being obtained from real‐life infection data, that is, the interactive map^16^ and BBC COVID‐19 in the UK website.^17^ The corresponding regression plots can be seen in Figures 1 and 2. In the real‐life LOPA tool application of this methodology, the population number and place of residence will not be random as it would depend on the chemical plant location, employees' numbers, and addresses.

**TABLE 1:** Data for areas around a Chemical Plant June 21, 2021

Data for areas around a Chemical Plant June 21, 2021		
Random population sample	Infection/case rate per 100,000 people	Area (home address)
21	207	East Renfrewshire
13	193	Renfrewshire
17	199	Argyll and Bute
39	214	North Ayrshire
52	279	East Ayrshire
9	169	South Ayrshire
6	106	Stirling
29	178	North Lanarkshire
30	235	East Dunbartonshire
23	163	Clackmannanshire
26	137	Angus
19	118	Fife
33	164	West Lothian
43	220	Perth and Kinross
76	392	East Lothian
36	197	South Lanarkshire
63	302	Midlothian
47	253	West Dunbartonshire
55	337	City of Edinburgh

**TABLE 2:** Data for areas around a Chemical Plant June 28, 2021

Data for areas around a Chemical Plant June 28, 2021		
Random population sample	Infection/case rate per 100,000 people	Area (home address)
21	430	East Renfrewshire
13	486	Renfrewshire
17	235	Argyll and Bute
39	250	North Ayrshire
52	455	East Ayrshire
9	247	South Ayrshire
6	269	Stirling
29	351	North Lanarkshire
30	432	East Dunbartonshire
23	277	Clackmannanshire
26	423	Angus
19	375	Fife
33	359	West Lothian
43	363	Perth and Kinross
76	625	East Lothian
36	279	South Lanarkshire
63	676	Midlothian
47	455	West Dunbartonshire
55	585	City of Edinburgh

The regression line equation from Figure 1 is y=3.4358x+98.651, making the geometrically adjusted mean infection rate value equal to 194.92. This is compared to the regression line from Figure 2, which is equal to y=4.9198x+233.59, making the geometrically adjusted mean infection rate value equal to 371.43. Refer to Appendix I, eqs. 1–4.

## 5 TRANSMISSION RATE CALCULATION

The transmission rate is calculated based on the infection rate obtained by regression analysis and eq. 5 in Appendix I. Tables 3 and 4, respectively, illustrate the calculation of transmission rate and COVID fatality Index for weeks 21.06.21 and 28.06.21, which are 18.14 and 34.56 per year. The increase was due to the virulent Delta strain emergence.

**TABLE 3:** Calculation for infection transmission rate—June 21, 2021

Infection in closed space office and open plant area	Case 1
Selected location	Chemical Plant
Date	June 21, 2021
Infection rate per 100,000	194.92
Infection rate as decimal per individual	0.0019492
Number of risk encounters per shift	50
Number of risk events per year	243.33
Percentage of chemical plant employees tested negative (Lateral Flow Testing)	0.70
Lateral flow testing is only 57.5% sensitive	0.40
Estimate for the asymptomatic proportion of SARS‐CoV‐2 infections is 28%	1.28
Infection Transmission Rate per Year	18.14

Calculation tables color key	
	User input
	Calculation output
	Input from other sources (relevant websites)
	Change variable

**TABLE 4:** Calculation for infection transmission rate—June 28, 2021

Infection in closed space office and open plant area	Case 2
Selected location	Chemical Plant
Date	June 28, 2021
Infection rate per 100,000	371.44
Infection rate as decimal per individual	0.0037144
Number of risk encounters per shift	50
Number of risk events per year	243.33
Percentage of chemical plant employees tested negative (lateral flow testing)	0.70
Lateral flow testing is only 57.5% sensitive	0.40
Estimate for the asymptomatic proportion of SARS‐CoV‐2 infections is 28%	1.28
Infection Transmission Rate per Year	34.56

*Note*: Please refer Table 3 for calculation tables color key.

## 6 IMPACT ON INDIVIDUAL'S RISK OF DEATH

ALAMA COVID‐19 Medical Risk Assessment^23^ defines the concept of COVID‐age as follows:

COVID‐age assesses an individual's vulnerability to COVID‐19 in the absence of previous infection or vaccination. This evidence indicates that vulnerability to COVID‐19 increases exponentially with age; for example, in comparison with a healthy person aged 20, a healthy person aged 60 has more than 30 times the risk of dying if they contract COVID‐19. COVID‐age summarizes vulnerability for combinations of risk factors, including age, sex, ethnicity, and various health problems. The final result of the COVID‐age calculator is the individual's risk of death, as a probability with their actual age.

The upper and lower fatality limits are calculated using the method presented by Coggon et al.^23^ per 1000 people infected (see eq. 4), and their geometric mean of a one person fatality is therefore evaluated and used for LOPA modeling, Table 5.

**TABLE 5:** Fatality calculation (COVID‐19 medical risk assessment—ALAMA, 2021)

General for all cases	Age actual, category	Sex	Ethnicity	BMI	Heath status	COVID age	Lower fatality limit	Upper fatality limit	Geometric mean of fatality for 1 person
A	62	Male	Asian	40+	Good	77	13	52	2.60E‐02
B	40	Male	White	30–34.9	Good	45	0.5	1.9	9.75E‐04
C	40	Female	White	30–34.9	Good	40	0.3	1.2	6.00E‐04
D	40	Male	White	40+	Asthma, Type 2 diabetes	85+	30	119	5.97E‐02

*Note*: Please refer Table 3 for calculation tables color key.

Abbreviation: BMI, body mass index.

For the seasonal flu death rate, LOPA calibration, a figure of annual one death in 10,000 population, or 0.0001 death per annum is used.^24^

## 7 THE CALCULATION OF THE HEALTH PROTOCOLS FAILURE PROBABILITY

The estimation of the PFD for these protocols is based on statistical surveys. A statistical survey is any structured inquiry designed to obtain aggregated data, which may be qualitative or quantitative, where the individual or corporate identities of the respondents are in themselves of little significance.

The survey methodology uses sampling of individual units from a population and associated techniques of survey collection. In the case of LOPA, in order to obtain the degree of public compliance with the COVID‐19 prevention protocols, observational surveys can be made on samples of employee units in the office building and chemical plant area.

Similar to process safety LOPA, for COVID‐19, the probability of failure of the IPLs also needs to be calculated. Table 6 (social distancing), Table 7 (face mask), and Table 8 (hand hygiene) illustrate a hypothetical survey undertaken to estimate the probability of failure on demand for COVID‐19 protective measures. Refer to eqs. 6 and 7 in Appendix I.

**TABLE 6:** Social distancing survey for Chemical Plant

Assume a Chemical Plant with 100 personnel sampling (observe people for 60 min a working day and record how many people observe social distancing within this survey period)		
Survey (observation)		
Week 1 (June 21, 2021) social distancing survey		
Day 1, first survey: 54 people out of 100 do not observe 2 m distance rule;		0.54
Day 2, second survey: 40 people out of 100 do not observe 2 m distance rule;		0.4
Day 3, third survey: 66 people out of 100 do not observe 2 m distance rule;		0.66
Geometric mean =	0.5224	Probability of failure for social distancing
Week 2 (June 28, 2021) social distancing survey		
Day 1, first survey: 44 people out of 100 do not observe 2 m distance rule;		0.44
Day 2, second survey: 90 people out of 100 do not observe 2 m distance rule;		0.9
Day 3, third survey: 16 people out of 100 do not observe 2 m distance rule;		0.16
Geometric mean =	0.3987	Probability of failure for social distancing

**TABLE 7:** Wearing face mask survey for Chemical Plant

Assume a Chemical Plant with 100 personnel sampling (observe people for 60 min a working day and record how many people observe wearing face mask within this survey period)		
Survey (observation)		
Week 1 (June 21, 2021) face mask survey		
Day 1, first survey: 58 people out of 100 do not fully wear face mask;		0.58
Day 2, second survey: 110 people out of 100 do not fully wear face mask;		1.1
Day 3, third survey: 72 people out of 100 do not fully wear face mask;		0.72
Geometric mean =	0.7716	Percentage people that do not fully wear face mask
Then, PFD of wearing mask protection is	0.2814	
Week 2 (June 28, 2021) face mask survey		
Day 1, first survey: 30 people out of 100 do not fully wear face mask;		0.3
Day 2, second survey: 70 people out of 100 do not fully wear face mask;		0.7
Day 3, third survey: 28 people out of 100 do not fully wear face mask;		0.28
Geometric mean =	0.3889	Percentage of people that do not fully wear face mask
Then, PFD of wearing mask protection is	0.1418	

*Note*: Sample PFD calculation for week 1 (June 21, 2021) face mask efficiency; TYPE B MASK; probability = 62%–65% protection; therefore PFDs = 0.38–0.35; geometric mean of the two protection probabilities (i.e., mask filtering efficiency) = 0.3647, thus, geometric mean “0.7716” of people chose not to wear mask x mask filtering efficiency “0.3647” type B = 0.2814 PFD.

Abbreviation: PFD, probability of failure on demand.

**TABLE 8:** Hand hygiene survey for Chemical Plant

Assume a Chemical Plant with 100 personnel sampling (observe people for 60 min a working day and record how many people observe hand hygiene within this survey period)			
Survey (observation) to be conducted by HSE department			
Week 1 (June 21, 2021) hand hygiene survey			
Day 1, first survey: 120 people out of 100 do not wash their hands;			1.2
Day 2, second survey: 90 people out of 100 do not wash their hands;			0.9
Day 3, third survey: 46 people out of 100 do not wash their hands;			0.46
Geometric mean =	0.7920		Probability of failure for hand hygiene
Week 2 (June 28, 2021) hand hygiene survey			
Day 1, first survey: 22 people out of 100 do not wash their hands;			0.22
Day 2, second survey: 60 people out of 100 do not wash their hands;			0.6
Day 3, third survey: 36 people out of 100 do not wash their hands;			0.36
Geometric mean =	0.3622		Probability of failure for hand hygiene

**FIGURE 3:**
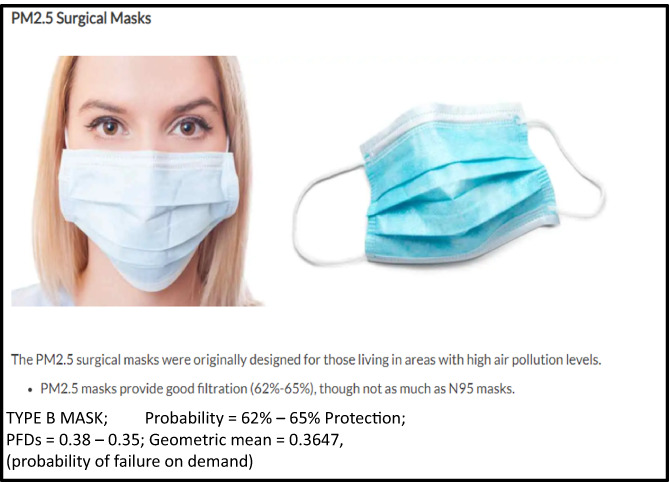
PM2.5 surgical mask independent protection layer calculation

There are many types of face masks that are commercially available. For this study, PM2.5 Surgical Masks are used, which are more widely used, as shown in Figure 3.

### 7.1 Ventilation

The main types of air cleaning that are likely to be effective at reducing infection risks include high‐efficiency particulate air (HEPA) filters and ultraviolet light.

According to Reference 25, the ventilation system factors that can minimize the virus spread are filtering, the number of air changes per hour (ACH) and recirculation. Based on ventilation design information, the following “rules” are proposed to estimate the ventilation system efficacy to combat virus spread.

- Rule 1; With or without recirculation, ACH > 12 with HEPA Filter or Equivalent PFD = 0.1
- Rule 2; No recirculation, ACH >6 with filter less efficiency than HEPA or Equivalent PFD = 0.5
- Rule 3; No recirculation, ACH <6 with filter less efficiency than HEPA or Equivalent PFD = 1.0

The recent study on ultraviolet light indicates that it can kill the new coronavirus. It is, however, challenging to assign an efficacy to this type of protection.

### 7.2 Vaccination

For vaccination efficacy, there are numerous sources of data depending on the type of vaccines, the real‐life data, and various interpretations of the results. Most vaccine manufacturers note efficacy above 90% and some between 70% and 80%; others go as low as 65%.^26^ However, it is emphasized that for all vaccine types, there is not any definitive efficacy figure. In order to use a conservative figure, based on considering possible breakthrough cases, this paper proposes to use 80% efficacy in order to estimate vaccination probability of failure.

## 8 RESULTS FOR CHEMICAL PLANT IN UNITED KINGDOM

**FIGURE 4:**
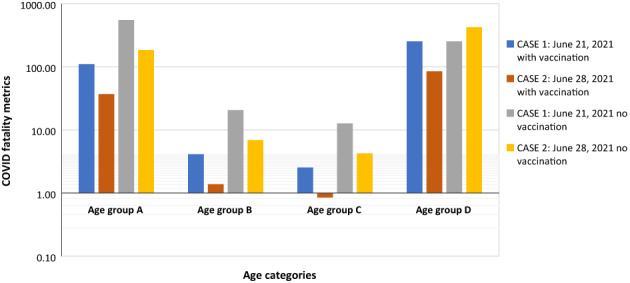
COVID fatality index for Cases 1 and 2, COVID fatality index is considerably lower for the vaccinated people (refer to Table 5 for category definition)

Tables 9 (with vaccination IPL) and 10 (no vaccination IPL) present LOPA calculation for the age group categories COVID fatality Indices for the two weekly periods. These LOPA Tables are constructed according to IEC 61511.^27^ The results of these LOPA calculations are the corresponding age group COVID fatality indices. As shown in Figure 4 for cases in Tables 9 and 10, the no vaccination age group has higher COVID fatality indices. Refer to eqs. 8–12 in Appendix I for LOPA calculation methodology.

**TABLE 9:** Layers of protection analysis and COVID fatality index calculation—June 21, 2021

Case 1, category (refer to Table 5 for category definition)	Infection transmission rate per year	Transmission pathway	Independent protection layers					Infection rate per year	Death probability for 1 infected person (refer to Table 5)	Risk of death per year	Flu annual death rate	COVID fatality index
			Social distancing	Building ventilation	Face mask	Hand hygiene	Vaccine					
A	18.14	Direct	0.5224	0.50	0.2814	0.7920	0.20	2.11E‐01				
	18.14	Indirect	0.5224	0.50	0.2814	0.7920	0.20	2.11E‐01				
		Total						4.22E‐01	2.60E‐02	1.10E‐02	1.00E‐04	109.80
B	18.14	Direct	0.5224	0.50	0.2814	0.7920	0.20	2.11E‐01				
	18.14	Indirect	0.5224	0.50	0.2814	0.7920	0.20	2.11E‐01				
		Total						4.22E‐01	9.75E‐04	4.12E‐04	1.00E‐04	4.12
C	18.14	Direct	0.5224	0.50	0.2814	0.7920	0.20	2.11E‐01				
	18.14	Indirect	0.5224	0.50	0.2814	0.7920	0.20	2.11E‐01				
		Total						4.22E‐01	6.00E‐04	2.53E‐04	1.00E‐04	2.53
D	18.14	Direct	0.5224	0.50	0.2814	0.7920	0.20	2.11E‐01				
	18.14	Indirect	0.5224	0.50	0.2814	0.7920	0.20	2.11E‐01				
		Total						4.22E‐01	5.97E‐02	2.52E‐02	1.00E‐04	252.33

*Note*: Please refer Table 3 for calculation tables color key.

**TABLE 10:** Layers of protection analysis and COVID fatality index calculation—June 28, 2021

Case 2, category case 1, category (refer to Table 5 for category definition)	Infection transmission rate per year	Transmission pathway	Independent protection layers					Infection rate per year	Death probability for 1 infected person (refer to Table 5)	Risk of death per year	Flu annual death rate	COVID fatality index
			Social distancing	Building ventilation	Face mask	Hand hygiene	Vaccine					
A	34.56	Direct	0.3987	0.50	0.1418	0.3622	0.20	7.08E‐02				
	34.56	Indirect	0.3987	0.50	0.1418	0.3622	0.20	7.08E‐02				
		Total						1.42E‐01	2.60E‐02	3.68E‐03	1.00E‐04	36.80
B	34.56	Direct	0.3987	0.50	0.1418	0.3622	0.20	7.08E‐02				
	34.56	Indirect	0.3987	0.50	0.1418	0.3622	0.20	7.08E‐02				
		Total						1.42E‐01	9.75E‐04	1.38E‐04	1.00E‐04	1.38
C	34.56	Direct	0.3987	0.50	0.1418	0.3622	0.20	7.08E‐02				
	34.56	Indirect	0.3987	0.50	0.1418	0.3622	0.20	7.08E‐02				
		Total						1.42E‐01	6.00E‐04	8.49E‐05	1.00E‐04	0.85
D	34.56	Direct	0.3987	0.50	0.1418	0.3622	0.20	7.08E‐02				
	34.56	Indirect	0.3987	0.50	0.1418	0.3622	0.20	7.08E‐02				
		Total						1.42E‐01	5.97E‐02	8.46E‐03	1.00E‐04	84.58

*Note*: Please refer Table 3 for calculation tables color key.

## 9 DISCUSSIONS

For chemical engineers, the usefulness of LOPA is obvious since this methodology appeared in the early 2000s, and it has become mandatory for any process design activity. LOPA reviews and calculations have become as important as hazard and operability reviews to design a safe and operable plant. In a sound chemical plant design, no process trip is designed without considering its safety integrity level requirement and its layers of protection (LOPA) implications. Based on the findings of this paper, it is proposed that the LOPA application to the COVID‐19 risk can also help to bring back some form of normality to the current pandemic situation.

Any risk can be mitigated depending on the availability and compliance of the safeguards, as well as the practicability of protective measures applications. This is the foundation of As Low As Reasonably Practicable, which is commonly used to design out the risks associated with process plants.

The permanency imperative of SARS‐COV‐2 would require that the COVID‐19 protection layer protocols and risk assessment tools have to be observed and implemented in some form. This paper aims to provide a scientific basis to apply the COVID‐19 protocols in the form of process safety engineering application of LOPA and get meaningful results to implement the safeguards efficiently. In order to achieve this aim, the level of public compliance to the COVID‐19 protective measures requires modeling and verification. The LOPA tool provides an algorithm to determine the steps that could be made to improve the situation, such as improving the infection encounter rate by reducing the number of contacts or events or setting up suitable testing regimes. There may be individuals who need special protection due to a combination of age, ethnicity, or BMI. They might be able to improve the effectiveness of COVID‐19 Protection Layer Protocols, either by physical improvements or encouraging compliance.

The LOPA tool enables the premises stakeholders to heed the public compliance of the COVID‐19 prevention safeguards and use the “COVID fatality Index” as a yardstick to devise plans and decisions to control and manage the virus spread amongst the population. The results indicate that LOPA can produce practical quantitative and qualitative results to be used for achieving and returning to a resilient normal life.

### 9.1 Conclusions

This study has demonstrated that LOPA is applicable to the COVID‐19 infection risk assessment. The LOPA tool presented here can be used to perform a “sensitivity analysis” by changing the input parameters and assessing the importance of these input variables to reduce the COVID fatality Index. The resulting decisions based on the LOPA sensitivity runs can develop plans, raise public awareness and communication for the public, devise testing plans, control the human encounter events, and develop more effective COVID‐19 protection layer health protocols.

This paper uses several calculation steps to reach the final results, which can be used for controlling the virus spread. If this methodology becomes automated, the users will not need to repeat all the calculation steps. Only the input parameters would need to be entered, and the automated tool would then generate a concluding figure that incorporates the COVID fatality Index, with some tabulated results. There are several input variables that can be changed in order to get the final outcome.

The advantage of this paper is that the main outcome is a calculated COVID fatality index value. The magnitude of this calculated value determines how much improvement in the transmission rate variables and the safeguarding protocols should be made in order to bring down the annual death rate in parity with the common flu. This Index can be used by stakeholders in the chemical industry to manage and control the spread of infection.

### 9.2 Recommendations

By now, it is well established that the SARS‐COV‐2 virus will be in circulation with variants in the human population permanently, as demonstrated by recent outbreaks of Omicron, Delta, or the original Wuhan strain.^28^, ^29^ The SARS‐COV‐2 infection must therefore be considered as a major hazard and should be treated similarly to a process plant, nuclear accident, or transportation risks. Process safety engineering has long demonstrated that probabilistic risk assessment and various qualitative risk reviews can reduce the process plants' accidental injuries and fatality risks.

It is, therefore, recommended that other process safety risk assessment tools may also be applied to the analysis of the COVID‐19 infection spread. Structured process safety reviews such as hazard identifications, with relevant modifications, may be applied to identify the COVID‐19 infection risks and make appropriate recommendations.

There are many other examples of process safety assessment tools that can potentially be applied to the pandemic situation, such as Performance Standards and Safety Critical Elements.^30^ In this case, it is concluded that efficient ventilation in closed spaces is the key to safeguarding against the virus spread in confined areas.^31^ The ventilation system can be treated as a safety critical element with the rigorous safeguarding performance standards as applied to process engineering critical equipment.

## AUTHOR CONTRIBUTIONS

**Ali Mokhber:** Conceptualization (lead); methodology (lead); software (equal); writing – original draft (equal). **Shivani Aggarwal:** Formal analysis (lead); writing – original draft (equal). **Pablo García‐Triñanes:** Project administration (lead); resources (lead); validation (equal); writing – review and editing (equal).

**Nomenclature**
ACH: air changes per hour
ALAMA: Association of Local Authority Medical Advisors
BMI: body mass index
IPLs: independent protection layers
LFTs: lateral flow tests
LOPA: Layers of protection analysis
PCR: polymerase chain reaction
PFD: probability of failure on demand

## Supporting information


**Data S1.** Supporting information.

## Data Availability

The data that support the findings of this study are available from the corresponding author upon reasonable request.
