# Supplementary material for: Application of layers of protection analysis to prevent coronavirus infection
Source: Process Safety Progress. 2022 Mar 31;41(3):469–79. doi: 10.1002/prs.12362 (PMC9111029; doi:10.1002/prs.12362)
Supplement: Supplementary file 1 — Data S1. Supporting information. [file PRS-41-469-s001.docx]

Application of Layers Of Protection Analysis to prevent coronavirus infection

Ali Mokhber, Shivani Aggarwal, Pablo García-Triñanes

Materials and Chemical Engineering Group, School of Engineering, University of Greenwich, Medway ME4 4TB, United Kingdom

[appendix I – Calculation Algorithm 3](#_Toc97482314)

# appendix I – Calculation Algorithm

Basis of calculations for various parameters are described as follows.

Regression Analysis and Statistical Modelling

Regression is used in statistical modelling, and it models the relationship between variables and their movement in the future. The method is widely used in the industry for predictive modelling and forecasting measures.

|  | $Y=a+bX$ | Eq. 1 |
| --- | --- | --- |

where:

*Y* = Dependent variable; Infection Rate per 100000 population

*X* = Independent variable; Random Population Sampling

*a* = The intercept

*b* = The slope

*n* = Number of observations

The formula for intercept “*a*” and the slope “*b*” can be calculated as per below.

|  | *a = (ΣY)(ΣX^2^) – (ΣX)(ΣXY)/ n(ΣX^2^) – (ΣX)^2^* | Eq. 2 |
| --- | --- | --- |

and

|  | *b = n (ΣXY) – (ΣX)(ΣY) /n(ΣX^2^) – (ΣX)^2^* | Eq. 3 |
| --- | --- | --- |

Geometric Mean Fatality Calculation

The mean arises when statistical averages must be corrected to compensate for data imbalances and large variances. The geometric mean equation is:

|  | $\left( F_{mean} \right)^{\frac{1}{n}}=\sqrt[n]{F_{1}F_{2}\ldots.F_{n}}$ | Eq. 4 |
| --- | --- | --- |

where *F_mean_* = Geometric mean fatality, *n* = Number of values and *F_i_* = Values to average.

Transmission Rate Calculation

This parameter is calculated by defining the following variables:

D = Rolling Infection rate per 100,000

D_1_ = Rolling Infection rate per individual; D/100000

D_2_ = Hours spent per day in risk areas

D_3_ = Total number of hours per year spent in risk areas

D_4_ = Total number of human transmissions per day

D_5_ = Percentage of population tested negative (Lateral Flow Testing)

D_6_ = Lateral flow testing is only 57.5% sensitive [18]

D_7_ = Estimate for the asymptomatic proportion of SARS-CoV-2 infections is 28% [19]

*T* = Infection Transmission Rate per Year

Thus:

|  | ${T=D}_{1}D_{2}D_{3}D_{4}\left( 1-D_{5}D_{6} \right)D_{7}$ | Eq. 5 |
| --- | --- | --- |

Risk management: Expert guidance - ALARP at a glance. https://www.hse.gov.uk/managing/theory/alarpglance.htm, 2021 [Accessed: 07/31/2021]

**IPL Probability Calculations for Health Protocols**

The IPLs probability of failure on demand is calculated by statistical observational surveys using below relationships:

|  | $P_{n=}\sum_{i=1}^{n} \frac{O_{i}}{O_{T}}$ | Eq. 6 |
| --- | --- | --- |
|  | $\left( P_{mean} \right)^{\frac{1}{n}}=\sqrt[n]{P_{1}P_{2}\ldots.P_{n}}$ | Eq. 7 |

where:

*O_i_* = Independent variable; observational survey

*O_T_* = Total number of independent variables; observational survey

*P_i_* = Values to average (dependent variable); IPL probability of failure on demand of health protocol

*n* = Number of values

*P_mean_* = Geometric mean of probability of failure on demand of health protocol

Basis of LOPA Calculations

The LOPA calculation is identical to process safety LOPA algorithms as follows:

|  | $I_{y1}=\sum TP_{1}P_{2}P_{3}P_{4}P_{5}$ | Eq. 8 |
| --- | --- | --- |
|  | $I_{y2}=\sum TP_{1}P_{2}P_{3}P_{4}P_{5}$ | Eq. 9 |
|  | $I_{y}=I_{y1}+I_{y2}$ | Eq. 10 |
|  | ${Covid}_{death risk}=I_{y}F_{mean}$ | Eq. 11 |

$$where:$$

*I_y1_* = Infection Rate per year (direct pathway of virus transmission)

*I_y2_* = Infection Rate per year (indirect pathway of virus transmission)

*I_y_* = Overall Infection Rate per year

*T* = Infection Transmission Rate per Year

*P_1_, P_2_, P_3_, P_4_ & P_5_* = Probability of failure on demand of health protocols (social distancing, ventilation, face mask, hand hygiene and vaccination)

*F_mean_* = Geometric mean fatality

COVID Fatality Index Calculation

The following formula was used for the calculation of the COVID fatality Index as a ratio between the COVID death risk and the annual flu death rate.

|  | $Covid Fatality Index=\frac{{Covid}_{death risk}}{Annual Flu Death Rate}$ | Eq. 12 |
| --- | --- | --- |
